# Two Years after the Fourth External Review: TDR Moves Forward with a New Vision and Strategy

**DOI:** 10.1371/journal.pntd.0000307

**Published:** 2008-11-25

**Authors:** Robert G. Ridley, Peter Ndumbe, Rolf Korte

**Affiliations:** 1 Special Programme for Research and Training in Tropical Diseases (TDR), World Health Organization, Geneva, Switzerland; 2 University of Buea, Molyko, Buea, Cameroon; 3 Institute of Hygiene and Environmental Health, Faculty of Medicine, Justus-Liebig-University, Giessen, Germany; *PLoS Neglected Tropical Diseases*, United States of America

TDR recently published an historical review of three decades of the organization's activities since its establishment as the Special Programme for Research and Training in Tropical Diseases in 1978 [1]. There have been four external reviews of TDR during this time, each followed by reorganization, institutional-fine-tuning, and adaptation to changing circumstances in the world of science and research. Independent external review has thus been integral to TDR, supporting its mission as a steward in forwarding public research interests, most particularly research on infectious diseases affecting the world's poorest and most disadvantaged populations.

The Fourth External Review [2], conducted between February 2005 and May 2006, was thus received as a core element of TDR's process of periodic institutional evaluation and adjustment. The findings of the review are summarized in an article in this issue of *PLoS Neglected Tropical Diseases* [3]. Ultimately, the review contributed to key elements of a new TDR Ten Year Strategy [4] and business plan [5] approved by TDR's Joint Coordinating Board in June 2007 and endorsed by the World Health Organization (WHO) (see Box 1). That strategy is now in the first stages of implementation.

**Box 1. TDR's New Ten Year Strategy**

**Vision:** *To foster an effective global research effort on infectious diseases of poverty in which disease-endemic countries play a pivotal role*.

**Key TDR Strategic Functions:**

- ***Stewardship*** for research on infectious diseases of poor populations: as facilitator and knowledge manager to support needs assessment, priority setting, progress analysis, and advocacy, and to provide a neutral platform for partners to discuss and harmonize their activities.

Top level objective is to bring about greater harmonization of global research efforts.

- ***Empowerment*** of researchers and public health professionals from disease-endemic countries, moving beyond traditional research training to build leadership at individual, institutional, and national levels so countries can better initiate and lead research activities, develop a stronger presence in international health research, and effectively use research results to inform policy and practice.

Top level objective is to promote disease-endemic country leadership in research.

- ***Research on neglected priority needs*** that are not adequately addressed by other partners. This will focus on three research functions:
  - Foster innovation for product discovery and development,
  - Foster research on development and evaluation of interventions in real-life settings,
  - Foster implementation research for access to interventions.

This strategic function will be managed through a limited number of well-defined and coherent activities termed business lines.

Top level objective is to bring about enhanced access to superior interventions.

## TDR's New Business Plan, in Light of the 2006 External Review

The Fourth External Review called for an increased emphasis on “needy populations” as compared to “neglected diseases” and a move towards a more trans-disciplinary view of health, defined by social as well as biomedical determinants. The new TDR vision statement (Box 1) reflects this recommendation. In practice, TDR is now less driven by the concept of a disease portfolio and more driven by infectious disease–related research needs and TDR's comparative advantage to address those needs.

**Figure 1:**
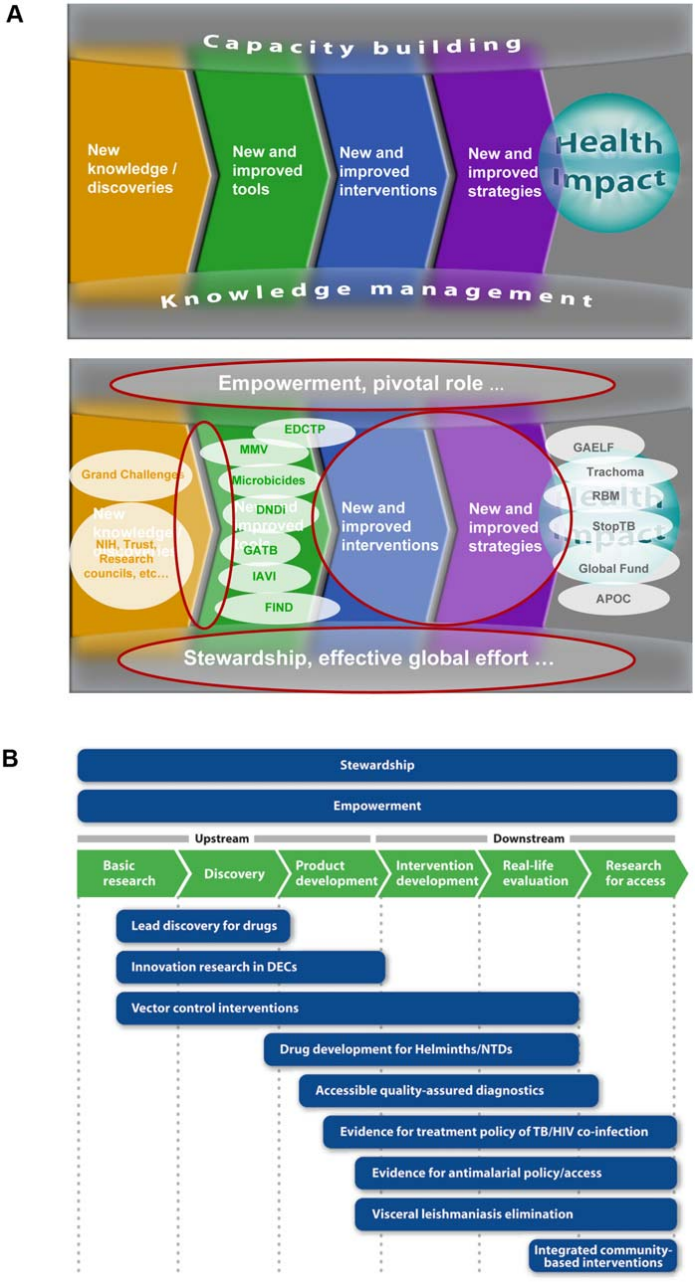
Analysis Using Health Research Continuum. (A) Functional assessment of health research continuum and gaps (in red) that need to be addressed. (B) Functional activities and business lines of new strategy mapped against the research continuum. Abbreviations: Grand Challenges, Grand Challenges in Global Health; NIH, US National Institutes of Health; Trust, Wellcome Trust; EDCTP, European and Developing Countries Clinical Trial Platform; MMV, Medicines for Malaria Venture; Microbicides, Alliance for Microbicide Development; DNDi, Drugs for Neglected Diseases Initiative; GATB, Global Alliance for TB Drug Development, IAVI, International AIDS Vaccine Initiative; FIND, Foundation for Innovative New Diagnostics; GAELF, Global Alliance for the Elimination of Lymphatic Filariasis; Trachoma, International Trachoma Initiative; RBM, Roll Back Malaria partnership; StopTB, Stop TB partnership; Global Fund, Global Fund to Fight AIDS, TB and Malaria; APOC, African Programme for Onchocerciasis Control. Image credit: WHO/TDR.

A diagrammatic representation of the analysis and functional manifestation of the new strategy is presented in Figure 1. Figure 1A illustrates the concept of a research continuum highlighting the cross-cutting features required to sustain relevant and high-quality research, namely knowledge management leading to the concept of stewardship, and capacity building leading to the concept of empowerment. The gap analysis identifies where TDR should place special emphasis in its research, specifically innovation for product development, intervention research, and implementation research for access to interventions.

Figure 1B illustrates how TDR's functions and business line research activities map out against the research continuum. Each of these activities is supported by an expert scientific advisory committee. The “business line” approach facilitates focused research efforts within a decentralized management and administrative structure that is responsive to change. Research business lines may be closed down as goals are reached or as activities are spun-off to other organizations, or initiated as new needs are identified through expert advice and stakeholder consultation, e.g., through its stewardship function, and endorsed through governance decisions.

In addition to the functional priorities of TDR discussed above, the Fourth External Review also made a number of recommendations relating to broad issues of governance, administration, and organization of the Programme. Before discussing those in more detail, we outline the overall process of TDR's strategy development and the role played by the Fourth External Review in that process.

## Process of TDR's Strategy Development

**Figure 2:**
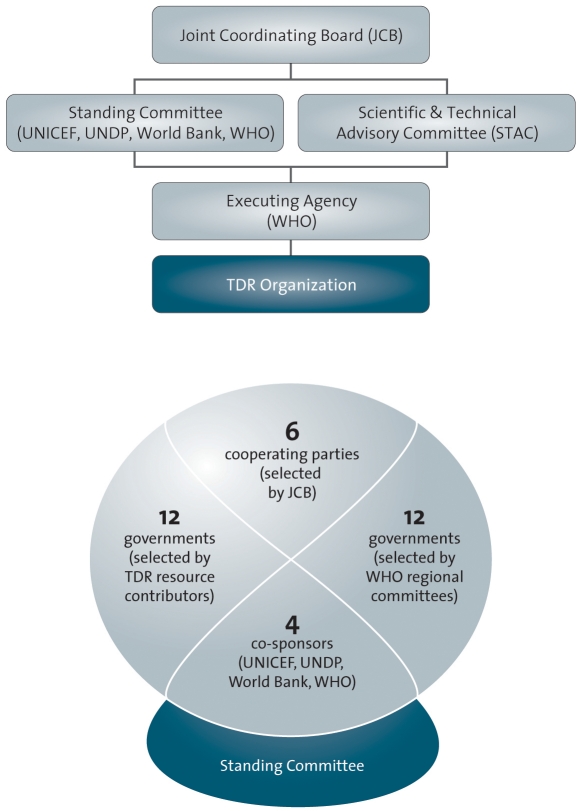
TDR Governance Structure. Image credit: WHO/TDR.

The process of TDR's strategy revision was dependent on its governance structure. This is depicted in Figure 2 [6]. TDR is governed by a Joint Coordinating Board (JCB) consisting of representatives of 30 national governments, equally divided between developed and developing countries, plus its four co-sponsoring agencies, the United Nations Children's Fund (UNICEF), the United Nations Development Programme (UNDP), the World Bank, and WHO. Its meetings are open to a wide range of observers that are identified as TDR cooperating parties and include representatives of additional national governments and representatives of academic and non-governmental institutions. TDR operates under the legal auspices of WHO as its executing agency. WHO is represented on the JCB through a special programme coordinator, normally an Assistant Director General to whom the Director of TDR reports. Two other significant bodies are (i) the Scientific and Technical Advisory Committee (STAC), which meets once a year to technically review the Programme and both advises the Director and reports to the JCB; (ii) the Standing Committee of the Joint Coordinating Board, which meets several times a year to monitor strategic and managerial issues in between the annual JCB meetings. The chairs of JCB and STAC also attend this committee meeting together with a JCB representative from both developed and developing countries.

The process of feeding in the comments of the Fourth External Review into the strategy development process is detailed in Box 2.

**Box 2. Process from Fourth External Review to New Strategy**

Initiation of Fourth External Review at request of 2004 JCB Jan 2005

Comment on Preliminary Report of Fourth External Review by STAC Feb 2006

Comment on Preliminary Report of Fourth External Review by Standing Committee March 2006

Final Report of Fourth External review May 2006

JCB assessment of Fourth External Review and comments by STAC and Standing Committee. Guidance provided on key directions for future strategy June 2006

Development of draft strategy document through external consultation and interim technical review by STAC Oct 2006

Special Stakeholder Consultation Meeting followed by Special Meeting of JCB to review draft strategy. Modifications suggested and request made for business plan to be developed Oct 2006

Review of draft business plan by STAC Feb 2007

Review of further draft business plan by Standing Committee March 2007

Review and final endorsement of business plan by JCB [7] June 2007

Formal initiation of strategy and business plan implementation within WHO Jan 2008

## Global Positioning of TDR: Role of Governance, Management and Administration

The first of the Fourth External Review's recommendations refers to the need for “ALL stakeholders to support TDR to evolve and grow to a renewed mandate that addresses the very neglected diseases and the health needs of the most needy populations”.

The revised TDR strategy and its implementation seeks to earn such support through being responsive to stakeholder issues through its stewardship, empowerment, and research functions. TDR's Joint Coordinating Board has also instituted several changes, with more under discussion, to make TDR's governance structures more responsive to its wider constituencies, notably within disease-endemic countries, to ensure that they play a “pivotal role” in TDR decision making. For example, consideration is being given to the participation of non-governmental constituencies on the Joint Coordinating Board. A useful summary of this discussion can be found in summary conclusions and recommendations number 53 to 60 of the Joint Coordinating Board Meeting of June 2007 [8].

A frequent complaint of organizations based at the UN relates to bureaucratic administrative processes, and the Fourth External Review expressed concern that TDR was over-administered and under-managed. The new management structure that forms part of the TDR business plan seeks to address this by decentralizing managerial and administrative authority within the Programme down to the level of the business lines. This is being complemented by a major initiative within WHO to improve its management and administrative practices, which will be of further benefit for TDR.

One of the recommendations from the Fourth External Review that was not taken up by TDR's governing bodies was the proposal to administratively decentralize its activities to regional centers. Following extensive discussion and debate, it was felt that while *functional decentralization* was seen as desirable, *administrative decentralization* would be counterproductive. Rather than making TDR more responsive to disease-endemic countries, it was feared that such a measure would increase costs and related bureaucracy, draining off resources that could otherwise be funnelled directly to developing country institutions, expert networks, and other groups.

Instead, key elements of the TDR business plan further these same aims without the creation of new TDR offices, for example through 1) increased emphasis on global and regional networks of experts and stakeholders; 2) creation of thematic and disease reference groups, to be hosted by different countries; 3) reinforcing relationships with the regional and country offices of WHO and other co-sponsoring agencies; and 4) support for the recruitment of individuals and consultants, based in disease-endemic country institutions, to facilitate TDR activities.

## Assessment of the Past and Looking Forward to the Future

TDR faced some serious issues at the time of the Fourth External Review and has responded to these through its new strategy and business plan. However, it is worth taking a deeper look at the extraordinary changes that were happening in global health research between 1999 and 2005 and the pressures these were placing on TDR. Perhaps the biggest single area of change was in the area of product development.

TDR's Third External Review in 1998 concluded that there was limited industry or public sector engagement in product development for neglected tropical diseases. TDR was thus asked to scale up its activities in this area and it responded. By the year 2000, TDR had roughly 15 product development activities underway, although many were chronically under-funded. Recognition by TDR's own governance and management that funding would be more effective through scaled up, externally leveraged and dedicated efforts led TDR to help conceptualize, incubate, and formally partner the establishment of several product development partnerships, notably Medicines for Malaria Venture (MMV) in 1999 and the Foundation for Innovative New Diagnostics (FIND) in 2003. TDR also significantly assisted in the development of the Global Alliance for TB Drug Development (TB Alliance) in 2000 and the Drugs for Neglected Diseases Initiative (DNDi) in 2002. TDR currently manages only two significant drug development projects. Some of the projects that were transitioned from TDR to product development partnerships (PPPs) during this period are listed in Box 3.

**Box 3. Projects Transitioned to Product Development Partnerships by TDR since 1999**

TDR-initiated projects transferred to new PPPs have included the following:

**MMV (1999 launch):** Pyronaridine plus artesunate; chlorporguanil-dapsone plus artesunate; dihydroartemisinin plus piperaquine; paediatric formulation of Coartem; synthetic peroxide.

**DNDi (2002 launch):** Amodiaquine plus artesunate; mefloquine plus artesunate.

**FIND (2003 launch):** Projects in TB and malaria.

**Institute for OneWorld Health:** Paromomycin for visceral leishmaniasis.

Such was the success of these initiatives, supported through new funding from both governments and philanthropic foundations such as the Bill & Melinda Gates Foundation, that by the time the Fourth External Review was issued, just eight years later, the pendulum had swung dramatically. Thus, a major conclusion was that TDR should significantly *reduce* the scope of its activities in these areas.

This kind of radical swing in focus places great pressure on an organization in terms of strategic direction, modes of operation, staff competencies, and commitment to different projects. Few organizations in this field could have transitioned so many of their own activities so quickly to other organizations, as did TDR over the past few years, in the name of advancing the broader goals of tropical disease research. The success of these transitions is testimony, again, to the inherent strengths and flexibility of TDR as an organization. From 1999 to 2006, TDR was also a partner in many research and capacity-building achievements (see Box 4); TDR progress reports covering these years can be found at [9]. TDR continued to generate through its funding approximately 250 peer-reviewed articles per year, with over 50% having a lead author from a developing country institution, combined with support for individual and institutional capacity strengthening.

**Box 4. TDR as a Partner in Research and Capacity Building, 1999–2006**

- Registration of miltefosine for treatment of visceral leishmaniasis with the Indian Council for Medical Research and Zentaris, leading to a political commitment to eliminate the disease from the Indian sub-continent by 2015.
- Label extension of Coartem (artemether–lumefantrine) for use in small children (5 kg), enabling its broader use, especially in Africa.
- Multi-country studies demonstrating the value of artemisinin-based combination therapy for malaria, leading to global policy recommendations.
- Network-based partnerships such as the Strategic Initiative for Developing Capacity for Ethical Review and the Forum for African Medical Editors.
- Facilitation of the sequencing of the mosquito genome and facilitation of a consortium for the sequencing of the tsetse fly genome.

We are not complacent about the issues that still face TDR. However, because TDR works through partners in a way that promotes their achievements, the achievements of TDR as an organization are often under-valued. TDR was judged by the Fourth External Review to be moderately successful from 1998 to 2005, largely due to the reduced global significance of the Programme with the arrival and excitement of new funds and new global initiatives. Paradoxically, we believe that when the history of this period is written, and particularly when judged against its budget, TDR's role will be seen to have been highly significant and successful in terms of its public health impact.

The one constant in this world is change. TDR, along with the rest of an increasingly well-resourced and vibrant health research community, needs to be continuously open to further adaptation and change. We need to manage such change in a way that coherently and sustainably advances a beneficial impact on people struggling to meet their health needs and the needs of their families and communities in conditions of poverty.

TDR is already making an impact in response to the gaps identified in the analysis outlined in Figure 1. Its plans for enhanced knowledge management and stakeholder consultation are on track with the recent launch of a new TropIKA web portal (http://www.tropika.net/) to promote these activities [10]. Networked initiatives with other institutions to promote disease-endemic country leadership in research are being initiated. In the area of translation research for product innovation, TDR has closely liaised with the WHO-led Intergovernmental Working Group on Public Health, Innovation and Intellectual Property [11], and TDR is establishing partnership networks to stimulate work in this area [12]. In the area of intervention and implementation research, TDR is spearheading new research processes that have demonstrated the power of community-directed interventions to address multiple diseases, including malaria, in an integrated way [13] and are influencing policy in Africa. TDR is also working with the Global Fund to Fight AIDS, TB and Malaria to promote frameworks for developing capacity for implementation and operational research [14].

The new strategy leaves TDR well placed to deal with future change.

It will achieve this if it remains true to its vision statement, inspired by many of the conclusions of the Fourth External Review, that its role is “to foster an effective global research effort on infectious diseases of poverty in which disease-endemic countries play a pivotal role”.
